# Luteolin suppresses UVB-induced photoageing by targeting JNK1 and p90^RSK2^

**DOI:** 10.1111/jcmm.12050

**Published:** 2013-04-04

**Authors:** Sung H Lim, Sung K Jung, Sanguine Byun, Eun J Lee, Jung A Hwang, Sang G Seo, Yeong A Kim, Jae G Yu, Ki W Lee, Hyong J Lee

**Affiliations:** a WCU Biomodulation Major, Center for Food and Bioconvergence, Department of Agricultural Biotechnology, Seoul National UniversitySeoul, Republic of Korea; b Functional Food Resources Research Group, Korea Food Research InstituteSeongnam, Republic of Korea; c Advanced Institute of Convergence Technology, Seoul National UniversitySuwon, Republic of Korea

**Keywords:** JNK1, RSK2, photoageing, luteolin, ultraviolet B (UVB)

## Abstract

Multiple lines of evidence suggest that natural compounds can prevent skin ageing induced by ultraviolet light. Luteolin, a bioactive compound found in chilli, onion, broccoli, celery and carrot, has been reported to exhibit anti-photoageing effects *in vitro*. However, the molecular targets and mechanisms of luteolin are still poorly understood. In this study, we sought to investigate the effects of luteolin on UVB-induced photoageing and the molecular mechanisms involved, using HaCaT human keratinocytes and SKH-1 hairless mice. Luteolin was found to inhibit UVB-induced MMP-1 expression in HaCaT cells, as well as UVB-induced activation of AP-1, a well-known transcription factor targeting the MMP-1 promoter region, as well as c-Fos and c-Jun, which comprise the AP-1 complex. In contrast, Western blot data showed that UVB-induced phosphorylation of JNK, ERK and p90RSK was not inhibited by luteolin. *In vitro* kinase assay data revealed that luteolin significantly suppressed JNK1 and p90RSK activity, but not that of JNK2 and ERK2. Pull-down assays showed that luteolin binds JNK1 in an ATP-competitive manner and p90RSK2 in an ATP-independent manner. Luteolin also inhibited UVB-induced wrinkle formation and MMP-13 expression, a rodent interstitial collagenase in mouse skin, *in vivo*. Taken together, our observations suggest that luteolin exhibits anti-photoageing effects *in vitro* and *in vivo* and may have potential as a treatment for the prevention of skin ageing.

## Introduction

Skin, is the most exposed organ of the body and in direct contact with sources of environmental stress including ultraviolet light (UV) 1. UV causes many detrimental physiological changes in the skin including epidermal inflammation, immunosuppression, hyperpigmentation, cancer and photoageing 2–5. UV can be divided into three types, according to wavelength; UVA (320–400 nm), UVB (280–320 nm) and UVC (200–280 nm). Although UVC has the highest energy, it is almost absorbed by the ozone layer, while UVA and UVB can reach the Earth's surface. UVB is the most biologically damaging UV type and significantly affects the epidermal layer of the skin, causing various skin disorders including premature skin ageing 6, 7. Although UV-induced skin ageing (photoageing) is a cumulative process and in part shares the same mechanisms as intrinsic ageing, photoageing relies entirely on the extent of UV exposure 1, 8.

The matrix metalloproteinases (MMPs) are a family of enzymes that can degrade components of the extracellular matrix (ECM). MMPs play important roles in morphogenesis, angiogenesis, arthritis, tumour invasion and photoageing 2, 9–11. MMP-1 (interstitial collagenase) plays the most prominent role in degrading native Type-1 and Type-3 collagen, major components of the skin dermis, while MMP-8 (neutrophil collagenase) and MMP-13 (collagenase-3) can cleave collagen to some extent. The cleavage of collagen by collagenases and the further degradation of cleaved collagen into gelatin and small peptides by gelatinases (MMP-2 and MMP-9) causes ECM breakdown, which is a major factor responsible for wrinkle formation 1, 4, 8. UVB irradiation-induced overexpression of MMP-1 is considered a biomarker, and its inhibition has been proposed to prevent wrinkle formation by stabilizing a balance between collagen degradation and synthesis 8, 12.

Previous studies have uncovered some of the molecular mechanisms underlying UV-induced MMP expression. UV activates growth factor receptors on the cell surface, including EGFR, followed by receptor-coupled signal transduction through pathways involving mitogen-activated protein kinases (MAPKs), Jak/STAT, protein kinase-C and PI3kinase/Akt 13–16. Activated kinases, including extracellular signal-regulated kinase (ERK) and c-Jun N-terminal kinase (JNK), then induce activation and expression of c-Jun and c-Fos, which are core components of the activator protein-1 (AP-1) complex. Jun proteins (c-Jun, Jun B and Jun D) and Fos proteins (c-Fos, Fos B, Fra-1 and Fra-2) homodimerize or heterodimerize to form the AP-1 complex, which is a transcription factor that binds to various target gene promoters including cyclin D1, cyclooxygenase-2 (COX-2) and MMPs including MMP-1. Increased AP-1 activity induced by UV plays a major role in the degradation of the ECM by activating transcription of MMP genes 2, 17–20.

**Fig. 1:**
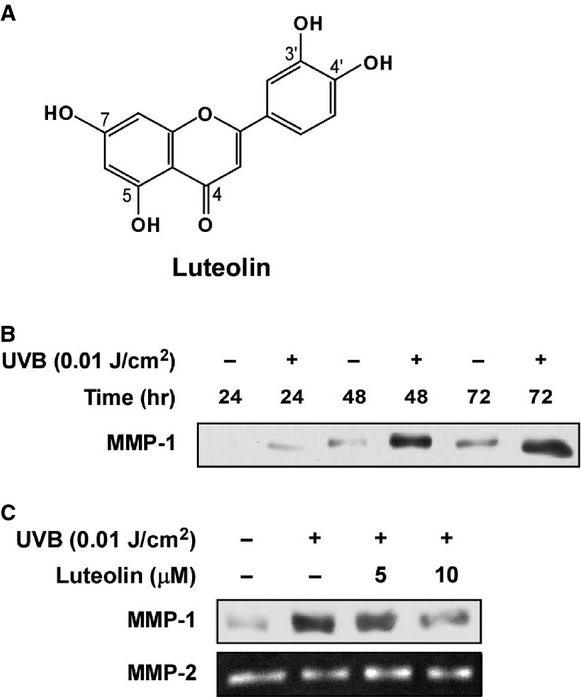
Effect of luteolin on UVB-induced MMP-1 expression in HaCaT human keratinocytes. (**A**) Structure of luteolin. (**B**) UVB induces MMP-1 expression in HaCaT cells. HaCaT cells were starved for 24 hrs in serum-free DMEM, washed with PBS and then irradiated with UVB (0.01 J/cm^2^) in a small volume of PBS. The cells were incubated for the indicated times and the level of MMP-1 expression in the culture media was determined by Western blot analysis using specific antibodies against the MMP-1 protein as described in Materials and Methods. Data are representative of three independent experiments that gave similar results. (**C**) Luteolin inhibits UVB-induced MMP-1 expression in HaCaT cells. HaCaT cells were starved for 24 hrs in serum-free DMEM and pretreated with luteolin at the indicated concentrations (0, 5, 10 μM). After 1 hr, the cells were washed with PBS and then irradiated with UVB (0.01 J/cm^2^) in a small volume of PBS. The cells were incubated for 48 hrs and the level of MMP-1 expression in the culture media was determined by Western blot analysis using specific antibodies against the MMP-1 protein as described in Materials and Methods. Data are representative of three independent experiments that gave similar results.

Luteolin (3, 4, 5, 7-tetrahydroxyflavone) is a flavonoid found in diverse fruits and vegetables such as chilli, onion, broccoli, celery and carrot (Fig. 1A) 21. It has been reported to exhibit antioxidant 22, 23, anti-cancer 24, 25, anti-inflammatory and anti-allergenic 26 effects. Moreover, recent studies have shown inhibitory effects of luteolin on UVA-induced MMP activation in human dermal fibroblasts 27 and on DMBA/TPA-induced skin tumorigenesis in a murine model 28. However, the molecular mechanisms and targets of luteolin in the prevention of photoageing remain poorly understood. Our objective was to examine the effects of luteolin on UVB-induced photoageing *in vitro* and *in vivo*, using HaCaT human keratinocytes and a mouse model of skin ageing.

## Materials and methods

### Materials

Luteolin (98%) was purchased from Indofine Chemical (Hillsborough, NJ, USA). Anti-human MMP-1 and antimouse MMP-13 antibodies were purchased from Epitomics (Burlingame, CA, USA) and Chemicon (Billerica, MA, USA) respectively. Antibodies to detect phosphorylated JNK (Thr183/Tyr185), total JNK, phosphorylated p90RSK (Thr359/Ser363), total p90RSK and phosphorylated c-Jun (Ser63) were purchased from Cell Signaling Biotechnology (Beverly, MA, USA). Antibodies against phosphorylated ERK1/2 (Thr202/Tyr204), total ERK, total c-Jun were obtained from Santa Cruz Biotechnology (Santa Cruz, CA, USA). Recombinant active JNK1 and JNK2 proteins were obtained from Upstate Biotechnology (Lake Placid, NY, USA) and recombinant active RSK2 was obtained from Signalchem (Richmond, BC, Canada). CNBr-Sepharose 4B, [γ-32P]ATP and the chemiluminescence detection kit were purchased from GE Healthcare (Piscataway, NJ, USA), and the protein assay kit was obtained from Bio-Rad Laboratories (Hercules, CA, USA). G418 and the luciferase assay substrate were purchased from Promega (Madison, WI, USA).

### Cell culture

The HaCaT human keratinocyte cell line was cultured in monolayers at 37°C in a 5% CO_2_ incubator in 10% FBS–DMEM with 2 mM l-glutamine and 25 μg/ml gentamicin. The HaCaT human keratinocyte cell line was stably transfected with the AP-1 and c-Fos luciferase reporter plasmid and maintained in 10% FBS–DMEM containing 200 μg/ml G418 29, 30.

### Animals

SKH-1 hairless mice (5 weeks of age; mean body weight, 25 g) were purchased from the Institute of Laboratory Animal Resources at Seoul National University (Seoul, Korea). Animals were acclimated for 1 week prior to the study and had free access to food and water. The animals were housed in climate-controlled quarters (24°C at 50% humidity) with a 12-hrs light/12-hrs dark cycle.

### Luciferase assay for AP-1 and c-Fos transactivation

Confluent monolayers of HaCaT cells stably transfected with the AP-1 or c-Fos luciferase plasmid were harvested, and 8 × 10^3^ viable cells were suspended in 100 μl of 10% FBS/DMEM and added to each well of a 96-well plate. Plates were incubated at 37°C in a 5% CO_2_ incubator. When cells reached 80–90% confluence, they were starved by culturing in serum-free FBS–DMEM for another 24 hrs. The cells were treated for 1 hr with luteolin (5 and 10 μM) before exposure to UVB (0.01 J/cm^2^), and then incubated for 5 or 3 hrs respectively. The cells were disrupted with 100 μl lysis buffer [0.1 μM potassium phosphate buffer (pH 7.8), 1% Triton X-100, 1 mM dithiothreitol (DTT) and 2 mM ethylenediamine tetraacetic acid (EDTA)] and luciferase activity was measured using a luminometer (Luminoskan Ascent; Thermo Electron, Helsinki, Finland).

### Western blot assays

Western blotting was performed as described previously 31. Cells were disrupted with lysis buffer [10 mM Tris (pH 7.5), 150 mM NaCl, 5 mM EDTA, 1% Triton X-100, 1 mM DTT, 0.1 mM PMSF, 10% glycerol and a protease inhibitor cocktail tablet] and the supernatant fractions were boiled for 5 min. Lysate protein (40 μg) was subjected to 10% sodium dodecyl sulfate–polyacrylamide gel electrophoresis (SDS-PAGE) and transferred to a polyvinylidene difluoride (PVDF) membrane (Amersham Pharmacia Biotech, Piscataway, NJ, USA). After transfer, the membrane was incubated with the specific primary antibodies at 4°C overnight. Protein bands were visualized using a chemiluminescence detection kit (Amersham Pharmacia Biotech) after incubation with a horseradish peroxidase (HRP)-conjugated secondary antibody.

For *in vivo* Western blotting, mice received topical application of luteolin (10 and 40 nmol) dissolved in 200 μl acetone on their backs 1 hr before UVB (0.18 J/cm^2^) irradiation. To isolate protein from mouse skin, the treated dorsal skin region of each mouse was excised and placed on ice. Fat was removed and the skin was placed in liquid nitrogen and immediately pulverized with a mortar and pestle. The pulverized skin was blended on ice with a homogenizer (IKA T10 basic; IKA Laboratory Equipment, Staufen, Germany), and skin lysates were centrifuged at 16,128 × *g* for 20 min. After the protein content was determined using a Bio-Rad protein assay kit, 100 μg of mouse skin lysate was subjected to 10% SDS-PAGE and transferred to a PVDF membrane (Amersham Pharmacia Biotech). Membranes were processed and proteins were analysed as described for the *in vitro* Western blot assay.

### Kinase assays

The *in vitro* JNK1 and JNK2 kinase assays were performed in accordance with the instructions provided by Millipore (Billerica, MA, USA). About 3 mM of the ATF2 substrate peptide was included. A 2.5 μl aliquot was removed from the reaction mixture containing 2.5 μl of each substrate and 10 μl diluted [γ-^32^P]ATP solution, and incubated at 30°C for 10 min. Fifteen microlitres aliquots were then transferred onto p81 paper and washed three times with 0.75% phosphoric acid for 5 min. per wash and once with acetone for 5 min. For RSK2, every reaction solution contained 25 μl of assay reaction buffer and a magnesium-ATP cocktail buffer. A 2.5 μl aliquot was removed from the reaction mixture containing 2.5 μl of each substrate and 10 μl diluted [γ-^32^P]ATP solution, and incubated at 30°C for 10 min.; then 15 μl aliquots were transferred onto p81 paper and washed three times with 0.75% phosphoric acid for 5 min. per wash and once with acetone for 5 min. The *in vitro* ERK2 kinase assays were performed in accordance with the instructions provided by Upstate Biotechnology. A total of 0.33 mg/ml of myelin basic protein substrate peptide was included. Four microlitres aliquots were removed after incubating the reaction mixture at 30°C for 15 min., to which 10 μl of diluted [γ-^32^P]ATP solution was added. This mixture was incubated for 10 min. at 30°C, and then 25 μl aliquots were transferred onto p81 paper and washed three times. The radioactive incorporation was determined using a scintillation counter. Each experiment was performed three times.

### Preparation of luteolin–Sepharose 4B

Luteolin–Sepharose 4B freeze-dried powder (0.3 g) was suspended in 1 mM HCl and coupled solution [0.1 M NaHCO_3_ (pH 8.3) and 0.5 M NaCl] was mixed. The mixture was rotated at 4°C overnight. The medium was transferred to 0.1 M Tris–HCl buffer (pH 8.0) and rotated end over end at 4°C overnight. The medium was washed three times with 0.1 M acetate buffer (pH 4.0) containing 0.5 M NaCl followed by a wash with 0.1 M Tris–HCl (pH 8.0) containing 0.5 M NaCl.

### Pull-down assays

Recombinant JNK1, JNK2 or RSK2 (0.1 μg) was incubated with luteolin–Sepharose 4B (or Sepharose 4B alone as a control) beads (100 μl, 50% slurry) in reaction buffer [50 mM Tris (pH 7.5), 5 mM EDTA, 150 mM NaCl, 1 mM DTT, 0.01% Nonidet P-40, 2 μg/ml bovine serum albumin, 0.02 mM PMSF and 1 μg protease inhibitor mixture]. After incubation with gentle rocking overnight at 4°C, the beads were washed five times with buffer [50 mM Tris (pH 7.5), 5 mM EDTA, 150 mM NaCl, 1 mM DTT, 0.01% Nonidet P-40 and 0.02 mM PMSF], and proteins bound to the beads were analysed by Western blotting**.**

### Mouse skin photoageing analysis

The animal experimental protocol (SNU-060512-1) was approved and experimental animals were maintained under specific pathogen-free conditions based on the guidelines established by the Animal Care and Use Committee of Seoul National University. Skin photoageing was induced in mice utilizing a UVB irradiation system. The UVB radiation source (Bio-Link crosslinker; Vilber Lourmat, Torcy, France) emitted at wavelengths of 254, 312 and 365 nm, with peak emission at 312 nm. SKH-1 mice were divided into four groups of three animals each. In the control group, the dorsal skin of the mice was topically treated with 200 μl acetone alone. In the UVB group, the dorsal skin of the mice was topically treated with 200 μl acetone 1 hr before UVB (0.18 J/cm^2^) irradiation. The mice in the third and fourth groups received topical application of luteolin (10 or 40 nmol) in 200 μl acetone 1 hr before UVB (0.18 J/cm^2^). These treatments were performed three times per week for 15 weeks.

### Grading of wrinkle formation

The evaluation of wrinkle formation was based on the method developed by Bissett *et al*., 32 with some modification. At 15 weeks after initiation of repeated UVB irradiation, the dorsal area of mice was photographed and three assessors (SHL, SKJ and SGB) blinded to the treatment conditions individually characterized the degree of wrinkle formation from the photographs of each animal, according to the grading scale describe in Table 1.

**Table 1:** Grading scheme for assessment of wrinkle formation

Grade	Description of wrinkling
0	No wrinkle
2	A few shallow wrinkles across back
4	Some coarse wrinkles across back
6	Several deep coarse wrinkles across back
8	Many deep, long wrinkles across back

### Statistical analysis

Where necessary, data are expressed as means ±SD, and student's *t*-test was used for statistical comparisons. A probability value of *P* < 0.05 and *P* < 0.01 was used as the criteria for statistical significance.

## Results

### Luteolin suppresses UVB-induced MMP-1 expression in human keratinocytes

To examine the effects of UVB radiation on MMP-1 expression in human keratinocytes, HaCaT cells were irradiated with UVB (0.01 J/cm^2^). MMP-1 levels in the culture media collected after the indicated durations were determined by Western blotting. UVB radiation induced MMP-1 expression after 48 hrs (Fig. 1B). Pretreatment with luteolin significantly suppressed UVB-induced MMP-1 expression in a dose-dependent manner (Fig. 1C).

### Luteolin inhibits UVB-induced AP-1 transactivation and c-Fos promoter activation in human keratinocytes

**Fig. 2:**
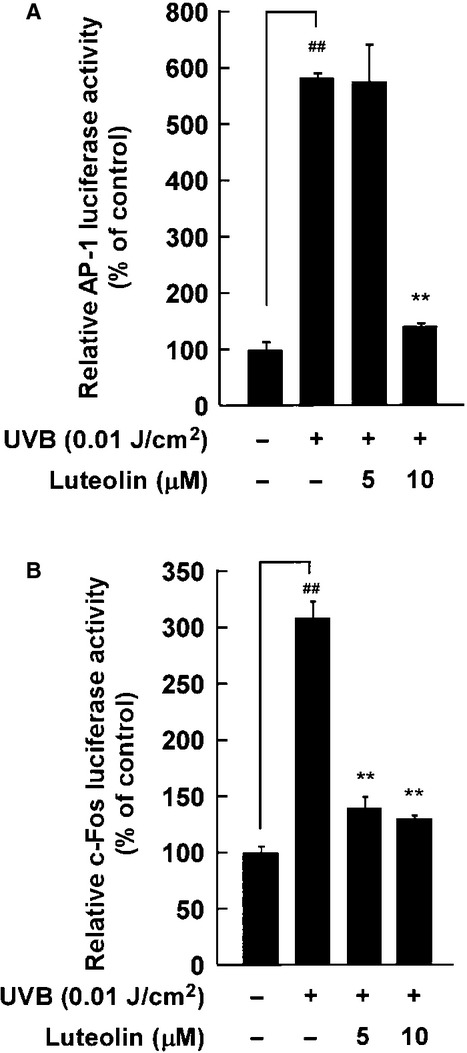
Effect of luteolin on UVB-induced AP-1 transactivation and c-Fos promoter activation in HaCaT human keratinocytes stably transfected with AP-1 and c-Fos luciferase reporter plasmids. (**A** and **B**) Luteolin inhibits both UVB-induced AP-1 transactivation and c-Fos promoter activation. For the luciferase assay, HaCaT cells stably transfected with AP-1 or c-Fos luciferase reporter plasmid were starved for 24 hrs in serum-free DMEM and pretreated with luteolin at the indicated concentrations (0, 5, 10 μM). After 1 hr, the cells were washed with PBS and then irradiated with UVB (0.01 J/cm^2^) in a small volume of PBS. The cells were then incubated for 5 hrs (A) or 3 hrs (B), and relative luciferase reporter activity was determined by analysing the cell lysates as described in Materials and Methods. AP-1 and c-Fos luciferase activities are expressed as the percent inhibition relative to non-UVB irradiated cells. Data are represented as the mean ±SD of AP-1 and c-Fos luciferase activity from three independent experiments (* and **significant differences at *P* < 0.05 and *P* < 0.01 between groups treated with UVB and luteolin and the group treated with UVB alone respectively).

UVB is known to induce Activator Protein-1 (AP-1) and *MMP-1* is a target gene with an AP-1 binding site in its promoter region 33, 34. The pretreatment of cells with luteolin inhibited UVB-induced AP-1 transactivation in HaCaT cells stably transfected with an AP-1 luciferase plasmid (Fig. 2A). We next investigated the effect of luteolin on UVB-induced c-Fos promoter activation. Pretreatment of cells with luteolin reduced UVB-induced c-Fos gene expression in HaCaT cells that were stably transfected with c-Fos luciferase plasmid (Fig. 2B).

### Luteolin suppresses UVB-induced phosphorylation of c-Jun in HaCaT human keratinocytes

**Fig. 3:**
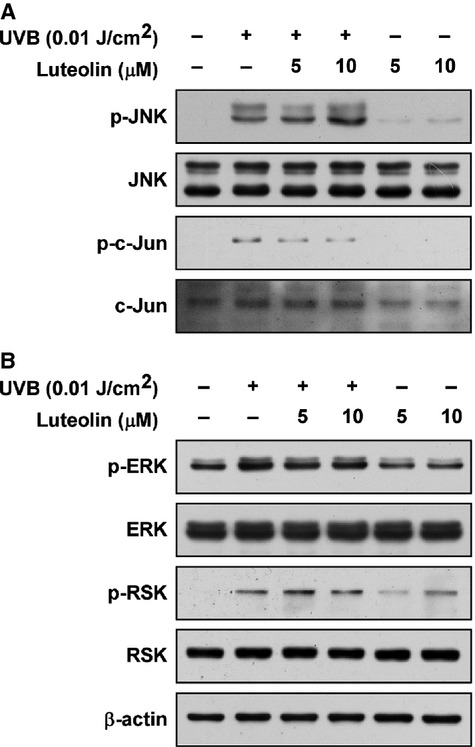
Effect of luteolin on UVB-induced JNK, c-Jun, ERK and RSK phosphorylation in HaCaT human keratinocytes. (**A**) Luteolin inhibits UVB-induced phosphorylation of c-Jun, but not JNK. (**B**) Luteolin does not inhibit UVB-induced phosphorylation of ERK, or p90RSK2. HaCaT cells were starved for 24 hrs in serum-free DMEM and pretreated with luteolin at the indicated concentrations (0, 5, 10 μM). After 1 hr, the cells were washed with PBS and then irradiated with UVB (0.01 J/cm^2^) in a small volume of PBS. The cells were incubated for 3 hrs and the levels of phosphorylated and total JNK, c-Jun, ERK and p90RSK protein were determined by Western blot analysis using specific antibodies against the corresponding phosphorylated and total proteins as described in Materials and Methods. Data are representative of three independent experiments that gave similar results.

In addition to c-Fos, activation of the AP-1 complex is dependent on phosphorylation of c-Jun, and we investigated whether luteolin has an effect on UVB-induced c-Jun phosphorylation. Pretreatment with luteolin significantly inhibited UVB-induced phosphorylation of c-Jun in HaCaT cells (Fig. 3A). However, luteolin exhibited no effect on UVB-induced phosphorylation of JNK (Fig. 3A), which is responsible for c-Jun phosphorylation. We then assessed the effect of luteolin on UVB-induced phosphorylation of ERK and p90RSK, the upstream kinases of c-Fos 35, 36. Pretreatment with luteolin had no effect on UVB-induced ERK and p90RSK phosphorylation in HaCaT cells (Fig. 3B).

### Luteolin inhibits JNK1 and p90RSK activities, but not JNK2 and ERK2 activities *in vitro*

**Fig. 4:**
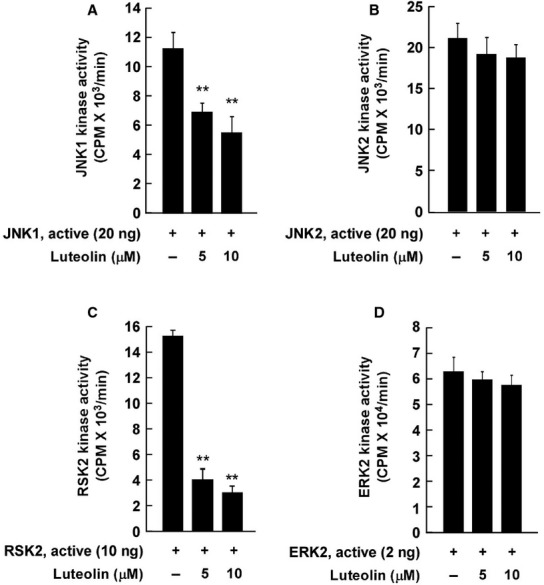
Effect of luteolin on JNK1, JNK2, p90RSK2 and ERK2 activities. (**A**) Luteolin inhibits JNK1 kinase activity. JNK1 kinase assays were performed as described in Materials and Methods. (**B**) Luteolin does not inhibit JNK2 kinase activity *in vitro*. (**C**) Luteolin inhibits p90RSK2 kinase activity *in vitro*. (**D**) Luteolin does not inhibit ERK2 kinase activity *in vitro*. Kinase assays were performed as described in Materials and Methods. Data are representative of three independent experiments that gave similar results. (**significant differences at *P* < 0.01, between groups treated with active kinase (JNK1, JNK2, p90RSK2 or ERK2) and luteolin, and the groups treated with active kinase alone).

Due to the fact that luteolin inhibited c-Jun phosphorylation, but not JNK phosphorylation (Fig. 3A), we suggested that luteolin might reduce c-Jun phosphorylation by inhibiting JNK activity through a direct interaction with JNK. *In vitro* kinase assay data shows that JNK1 activity was significantly suppressed by luteolin, but there was no significant change in JNK2 activity (Fig. 4A and B). Moreover, we investigated the effect of luteolin on p90RSK2 and ERK2 activity. The *in vitro* kinase assay data reveals that luteolin inhibits p90RSK2 activity, but has no effect on ERK2 activity (Fig. 4C and D).

### Luteolin binds JNK1 in an ATP-competitive manner and p90RSK2 in an ATP-independent manner

**Fig. 5:**
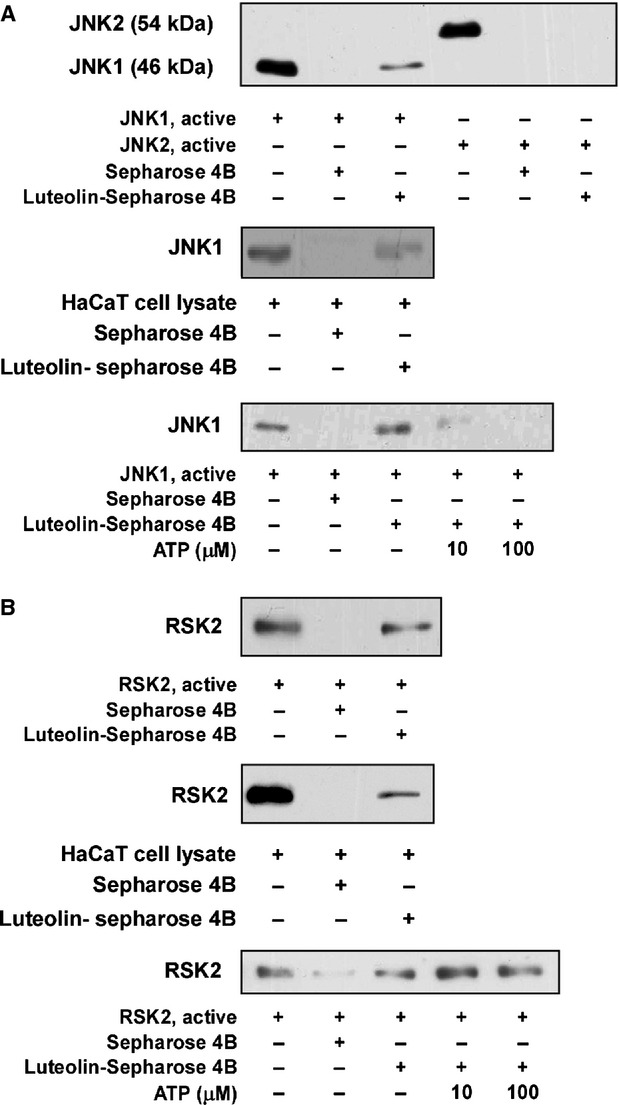
Binding affinity of luteolin with JNK1, JNK2 and p90RSK2. (**A**) Luteolin exhibits higher binding affinity to JNK1 than JNK2. Upper panel: *in vitro* luteolin binding was determined by immunoblotting with specific antibodies against JNK1 and JNK2 as described in Materials and Methods. *Lane 1*, active JNK1 (0.1 μg); *lane 2*, negative control, JNK1 does not bind with Sepharose 4B beads; *lane 3*, JNK1 binds with luteolin–Sepharose 4B beads; *Lane 4*, active JNK2 (0.1 μg); *lane 5*, negative control, JNK2 does not bind with Sepharose 4B beads; *lane 6*, JNK2 does not bind with luteolin–Sepharose 4B beads. Middle panel: *lane 1*, HaCaT cell lysate; *lane 2*, negative control, JNK1 does not bind with Sepharose 4B beads; *lane 3*, HaCaT cell lysate precipitated with Sepharose 4B beads. Lower panel: luteolin binds JNK1 in an ATP-dependent manner. *Lane 1*, active JNK1 (0.1 μg); *lane 2*, negative control, JNK1 does not bind with Sepharose 4B; *lane 3*, positive control, JNK1 binds with luteolin–Sepharose 4B; *lanes 4 and 5*, increasing concentrations of ATP suppress binding of luteolin to JNK1. (**B**) Luteolin directly binds with p90RSK2. Upper panel: *lane 1*, active p90RSK2 (0.1 μg); *lane 2*, negative control, p90RSK2 does not bind with Sepharose 4B beads; *lane 3*, p90RSK2 binds with luteolin–Sepharose 4B beads. Middle panel: *lane 1*, HaCaT cell lysates (0.1 μg); *lane 2*, negative control, p90RSK2 does not bind with Sepharose 4B beads; *lane 3*, p90RSK2 binds with luteolin–Sepharose 4B beads. Lower panel: luteolin binds with p90RSK2 in an ATP-independent manner. *Lane 1*, active p90RSK2 (0.1 μg); *lane 2*, negative control, p90RSK2 does not bind with Sepharose 4B beads; *lane 3*, positive control, p90RSK2 binds with luteolin–Sepharose 4B; *lanes 4 and 5*, increasing concentrations of ATP do not suppress luteolin binding with p90RSK2. Data are representative of three independent experiments that gave similar results.

*In vitro* and *ex vivo* pull-down assay indicates that JNK1 binds luteolin–Sepharose 4B beads (Fig. 5A, *upper and middle panels*). We further investigated the binding activity of luteolin with JNK1 in the presence of ATP. The binding activity of luteolin with JNK1 was reduced by ATP in a dose-dependent manner, suggesting that luteolin competes with ATP to bind JNK1 (Fig. 5A, *lower panel*). *In vitro* and *ex vivo* pull-down assays indicated that p90RSK2 binds luteolin–Sepharose 4B beads (Fig. 5B, *upper and middle panels*) and the binding activity of luteolin with p90RSK2 was not affected by the presence of ATP (Fig. 5B, *lower panel*), suggesting that luteolin suppresses p90RSK activity in an ATP-independent manner.

### Luteolin inhibits UVB-induced skin wrinkle formation in an SKH-1 hairless mouse model

**Fig. 6:**
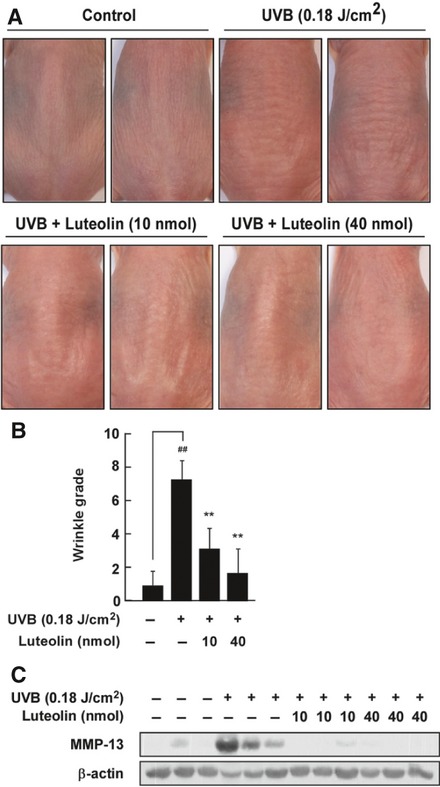
Luteolin inhibits UVB-induced wrinkle formation and MMP-13 expression in SKH-1 hairless mice. (**A**) Representative photographs showing anti-photoageing effects of luteolin. Mice were divided into four groups; controls treated with 200 μl acetone and non-irradiated; UVB group, treated with 200 μl acetone and UVB (0.18 J/cm^2^) irradiated; low dose sample group, treated with 10 μmol luteolin in 200 μl acetone and UVB irradiated; high dose sample group, treated with 40 μmol luteolin in 200 μl acetone and UVB irradiated. (**B**) Luteolin reduces UVB-induced wrinkle formation. The mice were treated as described for (A), and the extent of wrinkling was graded using a modification of the method proposed by Bissett *et al.,* 32. Data are represented as the mean ±SD (*n* = 3) (**significant differences at *P* < 0.01, between the UVB group and sample treated groups). (**C**) Luteolin inhibits UVB-induced MMP-13 expression. Protein was extracted from mouse dorsal skin as described in the Materials and Methods, and the level of MMP-13 expression was determined by Western blot analysis using specific antibodies against the MMP-13 protein.

To further evaluate the anti-photoageing effects of luteolin *in vivo*, we examined the outcome of treatment using a well-developed SKH-1 hairless mouse model 32. The dorsal skin of the mice was exposed to UVB (0.18 J/cm^2^) 1 hr after luteolin treatment, three times per week for 15 weeks. Representative photographs of the mice at the conclusion of the treatment duration show that luteolin inhibits UVB-induced wrinkle formation (Fig. 6A). The grade of wrinkle formation was quantified using a modification of the method proposed by Bissett *et al.,* 32 (Fig. 6B). To investigate the levels of collagenase in mice skin, we examined the expression of MMP-13, a rodent interstitial collagenase, using Western blotting of tumour cell lysates. Chronic UVB exposure induced MMP-13 overexpression, which was attenuated by luteolin treatment (Fig. 6C).

## Discussion

Our results demonstrate that luteolin suppresses UVB-induced MMP-1 expression in HaCaT cells, which may be a result of the direct inhibition of JNK1 and p90RSK2 activity. Luteolin also attenuated wrinkle formation induced by chronic UVB irradiation in SKH-1 hairless mice.

Skin ageing can be divided into two basic processes, intrinsic ageing and extrinsic ageing (photoageing) 8. While intrinsic ageing is characterized by smooth, dry, pale and finely wrinkled skin, photoageing is characterized by deep wrinkling and pigmentary changes, generally appearing on sun-exposed areas such as the face, neck and forearm 8. Ultraviolet radiation, especially UVB, is a major cause of photoageing and is known to induce MMP-1 expression in human dermal epidermis *in vivo* 2, as well as in cultured epidermal keratinocytes 37. MMP-1, an interstitial collagenase, also plays an important role by cleaving fibrillar collagens, which normally function to maintain skin elasticity and resilience. 3/4 N-terminal and 1/4 C-terminal triple-helical fragments are generated after cleavage reactions by collagenases (including MMP-1), which denature spontaneously into gelatin at body temperature and can be further degraded into smaller peptides by other MMPs 38. Therefore, the prevention or inhibition of MMP-1 overexpression induced by UVB could represent a novel strategy to reduce photoageing 12.

A number of previous studies have reported that natural compounds including luteolin exhibit anti-photoageing effects 18, 39, 40. However, the molecular mechanisms and targets of luteolin have not been clearly elucidated. Our result demonstrates that luteolin suppresses UVB-induced MMP-1 (Fig. 1C), as well as expression of AP-1, a well-known transcription factor that regulates MMP-1 gene transcription 41 and activation (Fig. 2A) in HaCaT cells. Because UVB-induced AP-1 activation was found to be inhibited by luteolin, we further investigated the phosphorylation and activation of c-Fos and c-Jun, which are core components of the AP-1 complex 33, using Western blotting and a luciferase reporter assay. UVB induced c-Fos promoter activation and c-Jun phosphorylation (Figs 2B and 3A), and these effects were inhibited by luteolin treatment. JNK, ERK and RSK are upstream kinases that regulate the transcription factors c-Fos and c-Jun 13, 35, 36. In contrast, UVB induced JNK, ERK and RSK phosphorylation, which was not inhibited by luteolin (Fig. 3A and B). We therefore hypothesized that luteolin could be directly inhibiting their kinase activity after phosphorylation. Our *in vitro* kinase assay results indicate that luteolin significantly inhibits JNK1 and p90RSK2 activity, but not that of JNK2 and ERK2. Furthermore, pull-down assays indicated that luteolin bound directly to JNK1 (in an ATP-competitive manner), but not JNK2 (Fig. 5A and B). Luteolin also bound RSK2 in an ATP-independent manner (Fig. 5C and D). Finally, we also determined that luteolin suppresses chronic UVB-induced wrinkle formation in SKH-1 hairless mice (Fig. 6A and B). UVB induced the expression of MMP-13, a rodent interstitial collagenase, in mouse skin lysates, an effect that was significantly reduced by luteolin treatment (Fig. 6C).

AP-1 is a well-known transcription factor that binds to the TPA-response element (TRE) with the consensus sequence TGACTCA and activates the transcription of target genes including MMP-1, MMP-3 and MMP-9 33, 34. The AP-1 complex consists of Jun and Fos family proteins that are known to be regulated by MAPKs 13, 29, 35. Phosphorylation of the c-Jun activation domain by JNK, a direct upstream kinase of c-Jun, activates its transcription activity 42. Together with c-Jun, c-Fos gene expression correlates with AP-1 activity 29 and is regulated by ERK, p90RSK and p38 35, 36. In this study, UVB irradiation was found to induce c-Jun phosphorylation and c-Fos activation, which was suppressed by luteolin, *via* direct inhibition of their upstream kinases (JNK1 and p90RSK2). p38 has also been reported to regulate UVB-induced c-Fos gene expression in HaCaT cells 13, but we saw no effect of luteolin on p38 phosphorylation (data not shown).

The c-Jun N-terminal kinases (JNKs) are members of the MAPK family and are heavily involved in apoptosis, cell survival and proliferation 43. JNKs can phosphorylate various AP-1 components, including c-Jun, JunD and activating transcription factor 2 (ATF2), and can also phosphorylate Elk1, c-Myc, p53, MLK2, tau and histone H3 43. In many cases, c-Jun has been reported to be phosphorylated only by JNKs. JNK1, JNK2 and JNK3 genes encode 10 isoforms of JNK, which are produced through alternative splicing of the genes 44. The role of JNKs in c-Jun phosphorylation remains controversial. Sabapathy *et al*., have reported that JNK2 is a negative regulator of c-Jun 45, whereas Jaeschke *et al*., contend that JNK2 is a positive regulator of c-Jun 46. Nevertheless, a consensus has been reached that JNK1 is a positive regulator of c-Jun 45, 46. In this study, we found that suppression of JNK1 activity by luteolin (Fig. 4A) is closely tied to inhibition of UVB-induced AP-1 activation. Further investigation into how luteolin differentially affects JNK1 and JNK2 is needed.

**Fig. 7:**
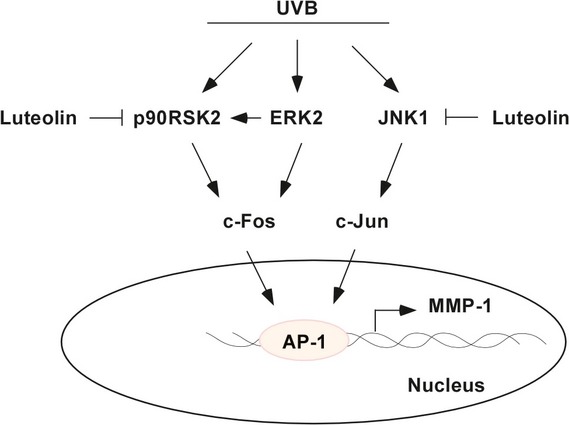
Simplified depiction of the proposed anti-photoageing mechanism induced by luteolin.

In summary, we have evaluated the preventive effects of luteolin on UVB-induced photoageing. Luteolin potently inhibited UVB-induced MMP-1 expression and wrinkle formation *in vitro* and *in vivo*. Our observations indicate that JNK1 and p90RSK2 are novel molecular targets of luteolin for the inhibition of UVB-induced signal transduction, leading to MMP-1 up-regulation (Fig. 7). We therefore conclude that luteolin may be useful as an agent for the prevention of UVB-induced skin ageing.
